# What are the barriers and facilitators to advance care planning with older people in long‐term care facilities? A qualitative study

**DOI:** 10.1111/jocn.17071

**Published:** 2024-02-20

**Authors:** Yuxin Zhou, Ariel Wang, Debbie Braybrook, Clare Ellis‐Smith, Haixia Feng, Ni Gong, Zhi Zhou, Richard Harding

**Affiliations:** ^1^ Cicely Saunders Institute of Palliative Care, Policy and Rehabilitation, Florence Nightingale Faculty of Nursing Midwifery & Palliative Care King's College London London UK; ^2^ Nuffield Department of Primary Care Health Sciences University of Oxford Oxford UK; ^3^ Department of Nursing Affiliated ZhongDa Hospital, School of Medicine, Southeast University Nanjing Jiangsu PR China; ^4^ School of Nursing Jinan University Guangzhou Guangdong PR China; ^5^ Department of Palliative Medicine, Nanjing BenQ Medical Center The Affiliated BenQ Hospital of Nanjing Medical University Nanjing Jiangsu PR China

**Keywords:** advance care planning, decision‐making, end‐of‐life care, long‐term care facilities, older people, palliative care

## Abstract

**Aim:**

To explore the views and preferences for advance care planning from the perspectives of residents, family members and healthcare professionals in long‐term care facilities.

**Design:**

A qualitative descriptive design.

**Methods:**

We conducted semi‐structured interviews with 12 residents of long‐term care facilities, 10 family members and 14 healthcare professionals. Data were analysed using reflexive thematic analysis. The social ecological model was used to develop implementation recommendations.

**Results:**

We constructed a conceptual model of barriers and facilitators to advance care planning in long‐term care facilities, drawing upon four dominant themes from the qualitative analysis: (1) The absence of discourse on end‐of‐life care: a lack of cultural climate to talk about death, the unspoken agreement to avoid conversations about death, and poor awareness of palliative care may hinder advance care planning initiation; (2) Relational decision‐making process is a dual factor affecting advance care planning engagement; (3) Low trust and ‘unsafe’ cultures: a lack of honest information sharing, risks of violating social expectations and damaging social relationships, and risks of legal consequences may hinder willingness to engage in advance care planning; (4) Meeting and respecting residents' psychosocial needs: these can be addressed by readiness assessment, initiating advance care planning in an informal and equal manner and involving social workers.

**Conclusion:**

Our findings show that residents' voices were not being heard. It is necessary to identify residents' spontaneous conversation triggers, articulate the value of advance care planning in light of the family's values and preferences, and respect residents' psychosocial needs to promote advance care planning in long‐term care facilities. Advance care planning may alleviate the decision‐making burden of offspring in nuclear families.

**Implications for clinical practice:**

The evidence‐based recommendations in this study will inform the implementation of context‐specific advance care planning in Asia‐Pacific regions.

**Patient and Public Contribution:**

Patients and caregivers contributed to the interview pilot and data collection.

**What does this paper contribute to the wider global clinical community?**

- Engagement with advance care planning can be improved by incorporating relational autonomy, such as identifying family dynamics and adapting involvement based on family decision‐making hierarchies and relationships.
- The complexity of the decision‐making process in long‐term care facilities suggests that advance care planning is an iterative and fluid process, which needs to be revisited in lockstep with changes in residents' physical and mental health status.
- It is imperative for nurses to serve as advocates and educators, capturing the signals of residents' spontaneous conversations to facilitate effective advance care planning and introducing palliation of suffering in response to the expression of a desire for a painless death.

## 1 INTRODUCTION

By 2050, China is predicted to have 25% of the world's older population (65 years or older) and the world's largest population of people aged 80 years or older (Luo et al., 2021). By then, 97 million older people are expected to live with disabilities, 36 million will have dementia and up to 60% of older people are expected to require daily care (Wang et al., 2019). The declining fertility rate in China, changes to family structures generated by the ‘one‐child’ policy, and the internal migration of young people have shifted the pattern of older care from the family to long‐term care facilities (Feng et al., 2020). Residents in long‐term care facilities commonly experience high levels of comorbidity and symptom burden, resulting in high dependency and clinical uncertainty (Pivodic et al., 2018; Stephens et al., 2018). The mean length of stay in such facilities for Chinese residents is 9 months, ranging from 3 to 12 months (Sun et al., 2020). Providing palliative and end‐of‐life care for older residents in long‐term care facilities is imperative.

Respecting an individual's preference for care and treatment is an essential component of high‐quality palliative and end‐of‐life care. However, delivering person‐centred care for older people is inherently challenging in Asia, where family‐dominated decision‐making remains the cultural norm while self‐determination is undervalued (Mori & Morita, 2020). Reflecting this, studies across Asia report that older people are typically not involved in decision‐making about their care (Ho et al., 2022; Lin, Evans, Koffman, Sheu, et al., 2019; Ozdemir et al., 2021).

## 2 BACKGROUND

Advance care planning is an iterative discussion process that enables individuals to identify their values and preferences for future care with family and healthcare providers (Rietjens et al., 2017). Advance care planning actively involves older people in their own care by encouraging the initiation of in‐depth discussions before they lose capacity to promote a shared understanding of their care preferences (Fleuren et al., 2020; Zhou et al., 2022). However, compared with other Asia‐Pacific countries and regions such as Singapore, Korea, Hong Kong and Taiwan (Cheng et al., 2020), advance care planning is a relatively new concept in mainland China. Currently in its infancy in terms of development, it is poorly understood by patients, professionals and the public, and there are a lack of localised programmes and national clinical practice guidelines to support implementation (Zhang et al., 2021).

Compared with community‐dwelling older people, residents in long‐term care facilities face fluctuating and uncertain disease trajectories, diminishing decision‐making capacities and functional abilities (Pivodic et al., 2018). Despite this, they do not appear to fear discussions around dying and have an interest in planning for end‐of‐life care (Zhang et al., 2015, 2021). The concept of advance care planning has originated largely from Western countries, and our recent review of evidence for the contextual factors that underpin advance care planning in long‐term care facilities found that research to date is Western‐centric (Zhou et al., 2022). Research has failed to consider how older people from non‐Western cultures make important end‐of‐life care decisions. The influence of concepts such as ‘filial piety’ which sets out certain obligations to older relatives, the importance of family‐centred decision‐making and the meaning of a good death in Asian communities have yet to be fully explored in relation to advance care planning (Zhou et al., 2022).

A recent integrative review synthesised current advance care planning research for older people in mainland China (Zhang et al., 2021). The review found that demographic factors such as gender and marriage were important influences on older peoples' attitudes to advance directives and advance care planning. However, findings from the review were limited by small, quantitative descriptive studies with cross‐sectional survey designs. They also focused on investigating the influencing factors of residents' attitudes towards written advance directive documents, rather than the process of iterative discussion which informs advance care planning. The mechanisms by which underlying cultural and social factors affect advance care planning in countries like China are uncertain and require further empirical investigation from a multiple stakeholder perspective. In Asia‐Pacific areas, there has been a rapid increase in the ageing population and thus the requirement for advance care planning, but there is limited evidence to inform development and implementation (Lin, Evans, Koffman, Armes, et al., 2019). Deepening our contextual knowledge of this area is paramount to developing successful advance care planning programmes for older people in long‐term care facilities in mainland China. This study therefore aimed to investigate views and preferences for advance care planning of residents, family members and healthcare professionals in Chinese long‐term care facilities. The specific objectives were to understand stakeholder experiences of decision‐making in long‐term care facilities and to explore potential facilitators and barriers to advance care planning in order to make recommendations for development and implementation.

## 3 METHODS

### 3.1 Study design and theoretical underpinning

This qualitative descriptive design study is part of a sequential multi‐methods research project to develop and preliminarily evaluate an advance care planning programme in long‐term care facilities in mainland China (Skivington et al., 2021). The study design is informed by the conceptual model derived from our realist review on the seven context‐mechanism‐outcome configurations that underpin advance care planning implementation in long‐term care facilities (Zhou et al., 2022). The study presented in this paper was guided by the critical realism paradigm, that is, the design acknowledges the ways individuals make meaning of their experiences and, in turn, the ways the broader social context influences those meanings (Fletcher, 2017). The study reporting followed the consolidated criteria for reporting qualitative research (COREQ) checklist (Tong et al., 2007) (See File S1).

### 3.2 Settings, participants and sampling

Long‐term care facilities in this study refer to facilities that have professional staff who provide 24‐hour nursing, rehabilitation and medical services (Feng et al., 2020). In China, over 50% of long‐term care facilities are privately owned (Feng et al., 2020). Four private facilities with 100–242 beds in Nanjing City, within Jiangsu province, were chosen based on existing research collaboration and geographical location. Recruitment, data collection and analysis were conducted iteratively until pragmatic saturation was reached, informed by the concept of information power (Braun & Clarke, 2021; Malterud et al., 2016). Inclusion criteria were (i) residents aged 65 years and over with the mental capacity to give informed consent; (ii) family members who acted as the primary carer(s) of residents (i.e. participate in the resident's care after admission) but not necessarily part of a dyad with family members; and (iii) healthcare professionals working at these study sites with experiences of caring for residents. Purposive sampling was applied to residents (by age, gender, education level and diagnosis), family members (by relationship to residents and education level) and healthcare professionals (by professional role, years of experience and whether trained in palliative care).

### 3.3 Recruitment

Residents, family members and healthcare professionals were identified, approached and recruited separately. The director of clinical nursing at each site introduced the study to eligible residents and family members by giving out the invitation letter and provided further information to eligible healthcare professionals at an informal meeting. The interviewer (YZ) visited those who expressed interest and were willing to share contact information, and subsequently provided detailed research information and participant information sheets. Participants were given at least 24 hours to consider study participation.

### 3.4 Data collection

Semi‐structured, face‐to‐face interviews were conducted by YZ, a female PhD student who has a background in nursing and training in both qualitative interviewing and advance care planning. The researcher was local to the study site, which enabled her to quickly establish rapport with participants and understand potential cultural cues and dynamics within interviews. Informed by a realist review (Zhou et al., 2022) and qualitative studies (Combes et al., 2021; Lin, Evans, Koffman, Sheu, et al., 2019), the topic guides included three areas: (i) experiences of end‐of‐life care communication; (ii) experiences in decision‐making on care at the end‐of‐life; (iii) views and preferences for advance care planning (see File S2). We used two case vignettes informed by a previous study (Combes et al., 2021) in the interviews to introduce advance care planning and facilitate discussion on sensitive topics by resonating with participants' lived experiences (see File S3). The topic guides and vignettes were refined after piloting the interview and checking with the patient and public involvement members and the supervisory team (CES, DE and RH). Interviews were conducted in a quiet room in the study sites to protect participants' privacy. All interviews were audio‐recorded. Reflective field notes were taken to capture researcher reflections and inform subsequent interviews.

### 3.5 Data analysis

**FIGURE 1:**
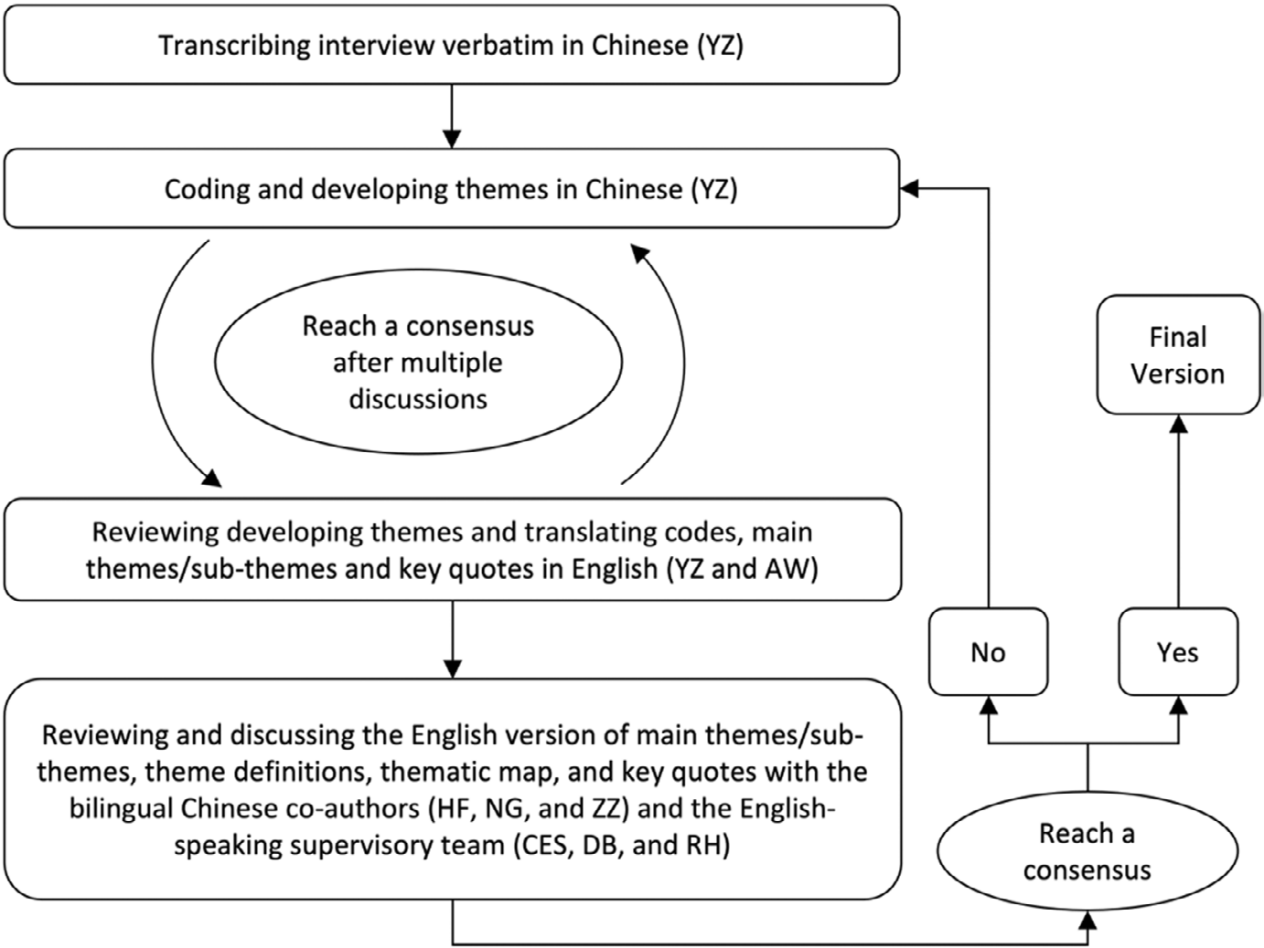
The study collaborative and iterative data analysis and translation procedure.

Interviews were transcribed verbatim using the software iFLYTEK and were also checked manually by YZ. We conducted inductively reflexive thematic analysis, that is, data familiarisation, systematic data coding, generating initial themes and reviewing and defining themes (Clarke & Braun, 2021; Tracy, 2010). All generated themes/sub‐themes, codes and key data extracts were checked by AW (a female bilingual researcher with experience in geriatric and qualitative research). They were then discussed with the supervisory team (CES, DE and RH) and bilingual Chinese researchers (HF, NG and ZZ). NVivo 12 was used to organise data, make memos and assist in coding and analysis. An iterative and collaborative analysis and translation procedure was adopted to ensure the study involved a multi‐faceted understanding of the phenomenon (Figure 1) (Douglas & Craig, 2007).

### 3.6 Rigour and reflexivity

The quality criteria we adopted to ensure the credibility and reflexivity of thematic analysis were listed in Table 1 (Clarke & Braun, 2021; Tracy, 2010).

**TABLE 1:** Adopted quality criteria for reflexive thematic analysis.

Quality criteria	Description
Rich rigour (the study uses appropriate theoretical constructs, samples, context, data and time)	We collected interviews from 36 stakeholders in four long‐term care facilities in China. The adequacy of sample size was monitored continuously during the process of data collection and analysis until pragmatic saturation was reached. Adequate time was allocated to complete the analytic process.
Sincerity (reflexive engagement and transparency)	Reflexive journaling was embedded throughout the research process to reflect on the researcher's (YZ) prior knowledge and assumptions on the context that may influence advance care planning in long‐term care facilities, personal experiences, subjective feelings to the data and sense‐making. Each phase of the analytic process is outlined transparently and clearly.
Credibility (thick description and crystallisation)	We built up a research team with different cultural backgrounds and multiple specialities (i.e. nursing, gerontology, epidemiology, anthropology and palliative care) to gain a more complex and in‐depth understanding of the data.
Resonance (transferability and naturalistic generalisation)	The data analysis is located within context, including the specific characteristics of residents, the long‐term care facilities and the Chinese and wider Asian sociocultural context. This maximises readers' potential to safely transfer the analysis to their context based on their understanding of the scene.
Meaningful coherence (analysis achieves its intended goals through using appropriate methods)	A previously conducted realist review (Zhou et al., 2022) supported the researchers' focus on exploring contextual factors underpin advance care planning in long‐term care facilities in China and informed the development of thematic guidelines.

### 3.7 Ethical considerations

This study received ethical approved from King's College London Research Ethics Committee (Ref: HR/DP‐20/21‐23,549) and the Independent Ethics Committee for Clinical Research of Zhongda Hospital, Affiliated to Southeast University (Ref: 2021ZDSYLL277‐P01). Written informed consents were obtained from all participants prior to the interview.

## 4 RESULTS

### 4.1 Sample characteristics

In total, 36 participants were interviewed between December 2021 and February 2022: 12 residents, 10 family members and 14 healthcare professionals (Table 2). The mean age of the residents was 83.8 years, and 60% of family members were daughters. More than half of the healthcare professionals were trained in palliative care. Of the 40 potential participants who were approached, four (one resident, three family members), declined to participate in the study due to their health conditions (*n* = 1) and being busy with the care of residents (*n* = 3). The mean interview duration was 48 min (range 19–77 min). Thirty‐two interviews were conducted independently (according to participant preferences), while two pairs of participants chose to be interviewed together, including a pair of siblings and two social workers.

**TABLE 2:** Sample characteristics (*N* = 36).

Characteristics of residents	*N* = 12	Characteristics of family members	*N* = 10	Characteristics of healthcare professionals	*N* = 14
Sex		Sex		Sex	
Male:female	5:7	Male:female	2:8	Male:female	5:9
Age (years)		Age		Age	
Mean (range)	83.8 (70–95)	Mean (range)	64.5	Mean (range)	37.6 (23–72)
			48–83		
Educational level		Relationship to residents		Professional role	
Primary school	2	Wife	2	Physician	4
Junior high school	3	Husband	1	Nurse	5
High school	4	Son	1	Therapist	2
University	3	Daughter	6	Social worker	3
Marital status		Educational level		Years of experience	
Married	4	High school	5	Median years (range)	10 (2–42)
Widowed	8	University	5	Trained in palliative care	
Comorbidity				Yes:no	6:8
Arthritis	4				
Diabetes	5				
Sleep disorders	3				
Osteoporosis	4				
Coronary heart disease	3				
Stroke	3				
Dementia	7				
Others (e.g. cancer, Guillain–Barre syndrome, pelvic fracture and cerebral haemorrhage)	5				

### 4.2 Thematic findings

Four dominant themes were constructed from the data: (1) The absence of discourse on end‐of‐life care; (2) relational decision‐making processes; (3) low trust and ‘unsafe’ cultures; (4) meeting and respecting residents' psychosocial needs.

#### 4.2.1 The absence of discourse on end‐of‐life care

##### Lack of cultural climate to talk about death

Family members reported that some members of the public in China hold misconceptions about death, believing it is a disease that can be controlled by medicine. Options such as refusing resuscitation were not highly valued by some participants. In these cases, they explained how advance care planning might be difficult to initiate because it is contrary to mainstream public beliefs:

The first challenge [of advance care planning] is the invisible pressure of traditional Chinese culture that as long as she (the patient) is still alive, you should do your best to treat illness with medicine and rescue the dying. it is still the majority's choice. I have heard people saying such as do not resuscitate and reduce suffering. They are not the Chinese mainstream. (F003: Male, husband)

In addition, healthcare professionals believed that discourse on end‐of‐life care remains scary or taboo due to misinformation regarding the idea of death. This resulted in increased challenges when initiating advance care planning:

The education we received when we were young was that death is a very scary thing. Especially for the older generation, they are afraid of death. That's why it's difficult for you to talk about death to them. (H006: Female, director of nursing)

##### Silence to protect each other from emotional distress

Unspoken agreement between residents and family members to avoid conversations about death further deprived residents of the opportunities to engage in advance care planning. Some professionals believed that discussing end‐of‐life care issues directly with residents might be interpreted as they were ‘giving up’ on them, be perceived as cruel and may increase their psychological distress. Therefore, in many cases, end‐of‐life care communication occurred only between family members and healthcare professionals:

The challenge [of advance care planning] is that residents may feel it's necessary [to join the end‐of‐life care discussion], but most of the time it is not possible [for the doctors and family members] to talk about this in front of them. When talking about end‐of‐life topics, they may think that you (doctors and families) are giving up on them. (H004: Female, nurse)

Some residents did spontaneously initiate conversations regarding preferred end‐of‐life care and after‐death arrangements. However, these conversations were sometimes interrupted by family members and healthcare professionals, who accused residents of introducing the topic of death prematurely. This led to residents feeling guilty and keeping silent as they realised that talking about their death increased the emotional distress of family members:

I can't say that [end‐of‐life care and after‐death arrangements]. When I say it, they will say, ‘Mom, stop saying that…’ I feel sorry to say that to them. (R004: Female, 91 years old)

##### Poor awareness of palliative care options

Seeking a death free from psychological suffering (by maintaining their dignity and not over‐burdening the family) and physical discomfort (by avoiding invasive treatments and managing pain) was raised by residents without prompting during the interview. However, some residents felt hopeless when they considered the prospect of losing their mental or physical functioning. Such feelings of hopelessness drove them to consider euthanasia, which they believed was the best or only way to freedom from pain. This belief could further limit their opportunities to initiate advance care planning and express care preferences:

Actually, I think euthanasia is better and what I fantasise about most is the night I die in my sleep. (YZ: What do you mean by euthanasia?) I think if someone feels hopeless about life, why bother? Simply passing away is the best. Because psychological pain is more painful than physical pain. (YZ: What is psychological pain?) If I ever lose my function… I don't want to put too much burden on my children. (R011: Male, 88 years old)

#### 4.2.2 Relational decision‐making processes

##### Shifting decision‐making responsibilities to the guardian: Inconsistencies in future care wishes

Healthcare professionals reported that conflict often arose when a family makes decisions, especially when residents have multiple children. Greater numbers of children increased the complexity of the decision‐making process and the risk of subsequent medical disputes. Therefore, long‐term care facilities routinely require families to nominate a ‘guardian’ (i.e. family representative) upon admission to serve as the proxy decision‐maker and family coordinator, despite residents still having capacity:

Many residents even have more than ten children. It is impossible to communicate with every one of them if there is any change in the resident's condition. Therefore, when the patient is admitted, we ask the family to choose a guardian, and we mainly communicate with the guardian. This is a way to avoid medical disputes. If there is a disagreement within the family, we still take the opinion of the guardian. (H001: Male, physician)

However, healthcare professionals explained how having a guardian did not always help them deliver goal‐concordant care, especially when these guardians were not aware of residents' wishes and preferences. In most cases, healthcare professionals explained how they attempted to balance a guardian's and resident's preferences for care. Such dilemmas helped them to recognise the value of identifying resident preferences prospectively through advance care planning:

Some of the guardians rarely come to visit or anything. Whether these guardians fully represent the patients' wishes? Maybe not always. It (advance care planning) is necessary. If the patient can participate, it will facilitate our understanding of their wishes. (H007: Female, physician)

In addition, some residents described their disinterest in planning for the future and were comfortable with their guardians making decisions for them. In these cases, advance care planning was not prioritised, and residents also considered planning as futile if guardians were not likely to comply with their wishes:

I don't care about these things (planning for future). I leave anything to the children to decide. I don't care, I don't ask, and I won't leave any last words. (YZ: Why?) It's useless if they don't follow [my wish]. (R006: Female, 95 years old)

Notably, participants spoke about the pressure of making proxy decisions in single child families. As one guardian reported, being the only child in the family meant that they had to bear the burden of making care decisions alone without the support of siblings:

Unfortunately, I'm the only child in my family, so all the burden is on me. I have to take the responsibility [of providing care] by myself and with no one else to discuss. You (the next generation) will certainly encounter this situation in the future. (F002: Female, daughter)

##### Valuing family relationships and family hierarchical structures

Healthcare professionals pointed out that family involvement is critical to the success of advance care planning given the importance of family, relative to the individual, in Chinese society. They recognised that advance care planning should be patient‐centred, but stressed that decisions should also consider complex and delicate relationships between residents and family members:

It (the resident's preferences) should account for a large proportion [in advance care planning], and then consider the nuanced emotions between him and his children to reach a consensus. Because in China, at any level, the family is a group, not an individual. (H003: Female, director of nursing)

Family members reported that the success of advance care planning may depend on the strength of relationships and harmony within the family:

In fact, I think that if there is no conflict in the family, the family meeting (advance care planning) will go smoothly. Because if the children in a family have harmonious relationships, there will be less conflict. (F005: Female, daughter)

Given family hierarchies, guardians (i.e. proxy decision‐makers) were generally the people who have the final say in the family. Therefore, sometimes, although other family members who were not identified as guardians were actively involved in the decision‐making process, they need to wait for the guardians to make a final decision:

We give him (the eldest son) the right to make decisions, we need to respect him, after all, he is our brother. Although he is not the person in charge. In Chinese traditional social norms, it is the son [who has the final say in the family]. (F005: Female, daughter)

##### Making timely and in‐the‐moment decisions

Given the unpredictable trajectories of residents' end‐of‐life illnesses, healthcare professionals recognised that advance care planning may help reach a consensus with residents and families prospectively, allowing timely decisions in the case of emergency. This would lead to reduced communication costs and delays in the treatment:

The difficulty [of end‐of‐life care decision‐making] is the sudden death of the elderly. In an emergency case during the night, we need to call the family first to discuss the treatment. [If there is an advance care planning] I can immediately deliver the treatment and don't need to constantly make phone calls. Because there is a risk that the repeated communication will delay the treatment for the residents. (H009: Female, head nurse)

Residents reported that making decisions about their future could be challenging and frightening, discouraging them from participating in advance care planning. Most of them expressed a preference for making shorter term or in‐the‐moment decisions, rather than planning ahead for an uncertain future:

I never think of this kind of [future] thing. It is difficult to think about things that haven't happened yet. I might pass out at any point, so I never think about it. All I need to worry about is my next meal. (R003: Female, 80 years old)

#### 4.2.3 Low trust and ‘unsafe’ cultures

##### Lack of honest information sharing

As the participating long‐term care facilities were privately owned, residents and family members suspected that some healthcare professionals were driven by profit. This meant that they did not always initiate honest discussions about disease prognosis and care planning with families. This led to inaccurate expectations for curative treatment and a lower awareness of the need for making advance care plans:

Doctors have to be honest about the prognosis and the survival time, not perfunctory and say it will be fine. When you (doctors) say it will be fine, of course I want to live, and my children will save me. (R005: Female, 74 years old)

##### Risks of violating social expectations and damaging social relationships

Family members feared that engaging in advance care planning would appear contrary to children's responsibility to do their best to ‘save’ their parents and might lead to judgement from others that they were being ‘unfilial’.

It's filial piety. Family members often tell us that although it (life‐sustaining treatments) costs them money and effort, [they will still choose it]. They fear being judged, such as the other relatives in the family and even neighbours who will blame them for not doing their best to save their parents behind their backs. (H005: Female, Therapist)

However, a daughter pointed out that key to the concept of filial piety (孝顺, ‘xiàoshùn’) in traditional Chinese culture is obedience (顺, ‘shùn’) to an older person's wishes, which is also a manifestation of filial (孝, ‘xiào’). Thus, the true essence of filial piety is to respect and fulfil the desire of older relatives, whatever that may be:

What is ‘filial piety’? Obedience is filial, filial is also obedience. If the elderly is willing to do this and he strongly expresses this desire, then children should have nothing to say. (F002: Female, daughter)

Residents also felt that if advance care planning provided an opportunity for residents to proactively and publicly discuss their wishes, it would help alleviate moral pressure on family members by easing the decision‐making responsibility:

I think [advance care planning] can reduce the pressure on children. People always think it's the children that give up therapy, they don't know that it's the elderly who doesn't want it. You have to have an open discussion to talk about this [elderly wishes]. (R002: Male, 89 years old)

Participants suggested promoting the potential value of advance care planning to older people and their families. Newer social norms could be acknowledged by communicating to families that advance care planning does not damage social relationships or contradict tradition:

Some people are afraid of being criticised by others. So let them know advance care planning is being advocated in society now and it's not something that goes against human relations and moral principles. (F001: Female, daughter)

##### Risks of legal consequences

Both healthcare professionals and family members were motivated to engage in advance care planning to reduce risk and protect themselves. Distrust and tense relationships with family members made healthcare professionals particularly concerned whether advance care planning would provide any legal protection, and whether they would become involved in medical disputes if family members chose to go back on their words:

Does this (advance care planning) conversation have a certain effect? For example, when the elderly is dying, can they (the family) take back or deny the content of [advance care planning] conversation? (H004: Female, nurse)

Even with formal guardianship appointments in long‐term care facilities, healthcare professionals were concerned about being accused of not resuscitating older residents by family members other than the guardian:

First of all, you have to regulate [advance care planning] legally. Although there is an appointed guardian, it is not always the guardian who comes to you when there is a conflict or problem. Other children also have the right to do so. (H006: Female, director of nursing)

Equally, some guardians themselves described the risk that advance care planning might cause in terms of family disputes if they make decisions which contradict the wishes of other members of the family:

Here's the paradox in China: your (residents') Wills cannot always determine the final decision since there are no laws stating that residents' Will is the final decision. Without legislation, we, the children [of residents] could be accused of murder. One of the children sues another [for murdering his parents without agreeing on the end‐of‐life care decision]. It can be very serious. (F002: Female, daughter)

Conversely, some participants recognised that advance care planning can be valuable by promoting consensus. This could reduce the likelihood that individuals within families would be blamed for making ‘wrong’ decisions by encouraging the sharing of decision‐making responsibilities:

I think it (advance care planning) should be encouraged. Because if you make a decision alone and it turns out to be bad, it will cause family disharmony. We make a decision together after discussion, even if it's wrong, nobody would be blamed. (F005: Female, daughter)

To remove potential legal risks, most participants agreed that advance care planning conversations should be documented and supported by national policy and laws, or notarised by a third party (e.g. Notary Public Offices) to regulate and monitor the actions of families and healthcare professionals:

It is best to have the doctor, the family and a notary [when making advance care planning]. If there is a third party, then everyone must follow this (advance care planning). The notary plays a supervisory role. (F008: Female, daughter)

#### 4.2.4 Meeting and respecting residents' psychosocial needs

##### Pre‐advance care planning readiness assessment

Healthcare professionals noted that it is necessary to assess residents' readiness for advance care planning as initiating it without preparation might increase psychological distress, particularly among those with high expectations of curative treatments:

For residents, it is important to find out whether they are willing to participate [advance care planning] because many of them have high expectations for survival. You need to select patients who have suffered from illness and hope to relieve suffering. (H002: Male, physician)

##### Initiating advance care planning in an informal and equal manner

Some residents held a positive attitude towards advance care planning, believing that it was a way to show them respect and for their wishes to be heard, acknowledged and memorised:

This (advance care planning) is evidence and the elderly's final decision. I think it also respects him most. It shows that people didn't forget him. What I need now is not sympathy, but respect. (R009: Female, 70 years old)

Family members and healthcare professionals were mindful of the sensitive, potentially distressing nature of advance care planning discussion. They suggested how it should be introduced to residents in a tentative, non‐threatening and indirect manner, making use of hypothetical scenarios of what can happen in different illnesses to improve engagement:

The challenge [of advance care planning] is that residents may be afraid to hear about the worst case. I think it (advance care planning) should be introduced using tentative language. For example, tentatively asking residents ‘Everything is going well for you now, how would you feel if things got worse?’(…)The best form is counselling. You can provide several hypothetical scenarios of common life‐limiting illnesses when an older person is admitted and ask about the family and his thoughts [on future care plan]. (F006: Female, daughter)

Family members noted that physicians often focused on the medical aspects of care during conversations but ignored residents' psychological needs and attitudes towards end‐of‐life care:

Doctors will emphasise more on the risks after the surgery, and they won't tell us the same thing (patient's and family's care preferences) you talked about. (YZ: What are the differences?) It's more humanised and more objective. Because you put the patient's attitude out there. (F006: Female, daughter)

In addition, social care professionals reported that the power imbalance between physicians and residents made their discussions excessively formal and increased the psychological distress of residents. Social workers were identified as the staff who could initiate advance care planning conversations in a more equal and informal way and bring psychosocial considerations into conversations:

Because the doctor seems quite authoritative, and if he is solemn, the resident may think this is very serious and need to make a big decision. But care assistants or social workers are different. We spend a long time together and we will talk to him like friends on an equal footing. (H013: Female, social worker)

Healthcare professionals recognised the importance of multidisciplinary collaboration in advance care planning to ensure that residents' physical and psychosocial needs are met. However, some suggested that medical staff with their busy schedules and a lack of training in communication skills should only be responsible for providing medical treatment. Initiating advance care planning conversations was seen as a role more suited to social workers. One nursing director suggested appointing trained staff to take on the responsibility of leading and coordinating advance care planning:

Because we (medical staff) are very busy, and we also lack [communication] training. We are responsible for medical care. Social workers shall take this responsibility [initiate advance care planning]. They could talk to the elderly and ask him [about his willingness to talk about end‐of‐life care]. It's better to have someone appointed to lead this (advance care planning). (H006: Female, director of nursing)

## 5 DISCUSSION

### 5.1 Summary of findings

**FIGURE 2:**
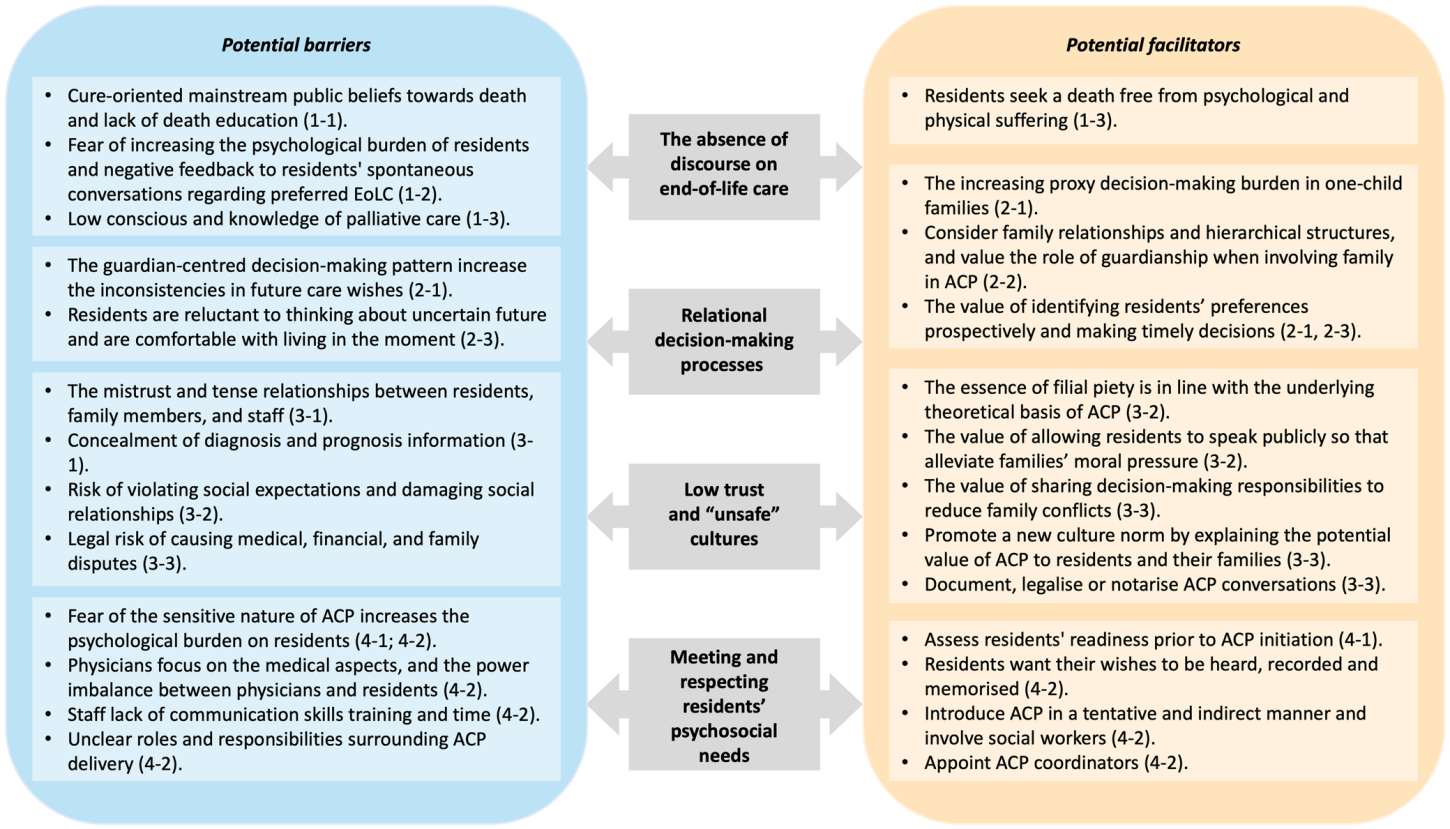
A conceptual model of potential facilitators and barriers to advance care planning in Chinese long‐term care facilities. ACP, advance care planning. [Colour figure can be viewed at wileyonlinelibrary.com]

This is the first qualitative study to identify the views and preferences for advance care planning in long‐term care facilities in mainland China from the perspectives of residents, family members and healthcare professionals. Our findings indicate that cultural norms can be a barrier to discussions about death and dying, and low awareness of palliative care options may hinder advance care planning initiation. Mistrust between residents/family and healthcare professionals and the perceived potential legal risks of participation strongly reduced willingness to engage in advance care planning. Furthermore, advance care planning in long‐term care facilities should recognise the value of family involvement, as well as the dynamic nature of decision‐making processes. Lastly, readiness assessment, initiating advance care planning in an informal and equitable manner, and involving social workers may increase resident engagement. Based on the findings, a conceptual model was constructed to show the potential facilitators and barriers to advance care planning in Chinese long‐term care facilities (Figure 2).

### 5.2 What this study adds

Our study found that the decision‐making pattern in long‐term care facilities was guardian‐centred and highly intertwined with family interests. Participants held an ambivalent attitude towards patient autonomy. They were aware of the importance of residents having the right to determine care at the end of their life. However, they also indicated that it was unrealistic to simply emphasise individual autonomy without taking into account the family and broader Chinese social context. This echoes the recent concern about the disconnect between the relational and social complexities of the decision‐making process and the individualistic philosophical underpinnings of advance care planning (Killackey et al., 2020). Our findings support the arguments that the engagement of stakeholders in advance care planning can be improved by incorporating relational autonomy, which recognises that self‐determination is formed and embedded in a social context (Killackey et al., 2020). Our residents and families reported that the benefits of advance care planning on family harmony and social relationships are the main factors influencing their attitudes. This suggests that in the Chinese context, promoting advance care planning as an individualistic project may not resonate emotionally and morally with the public.

However, as raised by participants, the essence of filial piety is to respect and fulfil the desire of older relatives. This is indeed in alignment with the underlying theoretical basis of advance care planning which respects individuals' wishes. It is likely that situating the values of advance care planning within the context of moral duties of the family in China, and reconceptualising advance care planning based on concepts such as filial piety would help to increase public acceptance. Family involvement was also considered a practice based on a relational understanding of autonomy. The importance of family involvement in advance care planning has been widely endorsed not only in Asia (Lin, Evans, Koffman, Sheu, et al., 2019), but also in parts of Europe and the United States (Kishino et al., 2022). Our findings highlight that in the context of long‐term care facilities, family involvement in advance care planning can be complex and varied. Healthcare professionals should identify family dynamics and how the family functions to increase engagement of residents and family members. This includes adapting to family relationships and hierarchical structures, as well as recognising the integrity of guardianship.

We found that the willingness of stakeholders to engage in advance care planning was also strongly influenced by the potential risks of legal consequences, and almost all stakeholders agreed with the importance of formally documenting advance care planning discussions. This result challenges the dominant view in the existing literature that advance care planning should focus on ‘process’ (discussion) rather than ‘outcome’ (documents) (Miyashita et al., 2022). Where there are low levels of trust between residents, family members and healthcare professionals in long‐term care facilities, advance care planning documents may be particularly valued.

Currently, there is no national guideline for advance care planning available in mainland China, and it has to date been promoted as part of the ‘living will’ (Zhang, 2021). In June 2022, the area of Shenzhen pioneered the legalisation of the living will to empower patients with autonomy (see File S4) (Shenzhen Municipal People's Congress, 2022). The living will highlights the importance of advance care planning in discussing and identifying patients' values and preferences. In 2017, the Adult Voluntary Guardianship Law in China's Civil Code permitted older adults to appoint a preferred guardian to make proxy healthcare decisions in their best interests, should they lose capacity (The National People's Congress of the People's Republic of China, 2017). It is necessary to raise awareness and promote implementation of these relevant laws and policies in long‐term care facilities to remove potential family and medical disputes and improve the acceptance of advance care planning.

In our study, some medical staff were reluctant to take responsibility for initiating advance care planning discussion and saw this as a role for social workers. Our recent review also identified the benefits of involving social workers in advance care planning to identify psychosocial needs and provide support (Zhou et al., 2022). However, how best to involve social workers in the process of advance care planning remains unclear. Social workers may be ideally placed and qualified to serve as coordinators, taking responsibility for initiating and organising advance care planning discussions and building rapport between residents, families and medical staff. It should also be noted that the importance of a multidisciplinary team is often overlooked in advance care planning practice, especially when it is assumed that a sole professional should take on this responsibility (Beck et al., 2017). It is essential to establish a trained multidisciplinary team with clearly defined roles and responsibilities not only to meet residents' psychosocial needs but also to provide medical care which is concordant with advance care plans (Rietjens et al., 2017).

Our findings highlight that despite living within a culture averse to conversations about death and dying, Chinese residents in long‐term care often spontaneously open conversations to express their desire for a painless death. This has been discussed in the literature and has been considered by some to be a manifestation of support for euthanasia among older people (Lei et al., 2022). However, our study suggests that a potential driver of this phenomenon is poor knowledge of the range of palliative care options available, perhaps reflecting the slow adoption of palliative care more generally in China (Yan et al., 2020). In addition, this low awareness among residents, allows the promotion of a myth that advance care planning will result in undertreatment or signals that death is imminent. Nurses, as the staff members who interact most with residents, are best placed to act as advocates when residents' voices are not being heard. When residents express wishes and preferences for end‐of‐life care, it is critical that nurses be able to capture such spontaneous conversation triggers, communicate their requests to family members and other healthcare providers, and facilitate effective advance care planning in a timely manner. In addition, nurses should be trained as educators to clarify misconceptions between palliative care and euthanasia, and to introduce palliative care when residents express their desire for a painless death.

There has been an intense debate over the value of advance care planning recently, with some researchers calling for a rethink of its purpose (Curtis, 2021). Our findings revealed the value of advance care planning to reduce the decision‐making burden of offspring in nuclear families. Although causing family conflict is a reported barrier to advance care planning in multiple‐child families, siblings can also provide mutual support and share the burden of proxy decision‐making for children who act as guardians. Moreover, influenced by China's one‐child policy (1980–2015), the responsibility for proxy decision‐making will shift entirely to the one‐child generation. Advance care planning which helps residents proactively discuss their values with their offspring may help to relieve the heavy burden of decision‐making for the only child.

### 5.3 Strengths and limitations

Our study contributes to the literature on the potential facilitators and barriers to advance care planning in mainland China by incorporating the perspectives of residents, family members and healthcare professionals. In line with the Medical Research Council framework for complex intervention development, this study also furthers the findings from our realist review to elucidate the context in which the advance care planning can be normalised into routine practice in long‐term care facilities (Zhou et al., 2022). We acknowledge that this study has limitations. First, unavoidable translation bias may increase the difficulty in interpreting the culturally situated meanings of participants. To maximise the data credibility, we adopted an iterative and collaborative reflexive data analysis and translation procedure. This approach, combined with study team members from different cultural backgrounds and specialities was used to address challenges of conceptual equivalence and cross‐cultural translation (Douglas & Craig, 2007). Second, because advance care planning is a new concept in Chinese society, we used two case vignettes and alternative terms (e.g. end‐of‐life care discussion and family meetings) to facilitate the interviews. This may introduce researchers' preconceptions about advance care planning and increase the bias regarding participants' real perceptions of the study topics (Lin, Evans, Koffman, Sheu, et al., 2019). However, we piloted and refined the vignettes and alternative terms, and then they were reviewed by patient and public involvement members and the supervisory team to ensure suitability and relevance for use in long‐term facilities in China. Last, we only sampled participants from private long‐term care facilities in one city, which may lead to sample selection bias. To ensure a suitable breadth of insights, further research is needed to explore differences in stakeholders' perceptions and preferences across different types of long‐term care facilities and geographical areas.

### 5.4 Implications for further research and practice

The Medical Research Council framework for complex interventions suggest considering implementation of interventions as part of the development phase (Skivington et al., 2021). Bradshaw et al. suggest that a socioecological lens that recognises the multi‐level factors impacting advance care planning implementation is beneficial (Bradshaw et al., 2021). In line with the four themes from our analysis and the Social Ecological Model (Mcleroy et al., 1988), we have developed recommendations for multi‐level implementation considerations to inform future advance care planning development in long‐term care facilities (Table 3).

**TABLE 3:** Multi‐level implementation considerations for advance care planning in long‐term care facilities based on themes.

Levels	Who	Considerations	Supported themes
System	Policymakers/organisations	Providing education among the public to improve death literacy.	Theme 1–1
		Raising awareness of ACP among the general public by interpreting the concept of ACP in line with social norms and articulating the value of ACP in light of the family's values and preferences.	Theme 1–1; 3–2
		Creating a ‘safe’ climate to increase ACP engagement. This includes advocating for laws and policies, as well as facilitating and promoting ACP with legal support from external agencies.	Theme 3–3
Cultural	Healthcare providers/organisations	Introducing ACP in an informal, tentative and indirect manner, such as using open‐ended questions, providing hypothetical scenarios and consulting.	Theme 4–2
		Adapting family involvement based on family authority hierarchies and relationships. Recognising the integrity of guardianship.	Theme 2–1; 2–2
		Assessing residents' readiness prior to ACP initiation. Possible readiness influencing factors include socioeconomic status, life‐and‐death attitudes, family background, mental capacity and mental health. Initiating ACP at a closer time to deterioration or death is more appropriate to increase ACP readiness.	Theme 4–1
		Promoting person‐centred and shared decision‐making to ensure that residents' voices are heard and the decision‐making burden on proxies can be eased.	Theme 2–1; 2–2; 4–2
Organisational	Healthcare providers/organisations	Normalising the topic of death and dying into daily clinical practice.	Theme 1–2
		Appointing ACP coordinators to build rapport between residents, family members and medical staff and organise ACP activities.	Theme 4–2
		Providing communication skills and ACP training and mentoring.	Theme 1–1; 4–2
		Establishing a multidisciplinary ACP team with clearly defined roles and responsibilities. Involving social workers to initiate ACP in a more equal and friendly manner.	Theme 4–2
Interpersonal	Healthcare providers/organisations	Identifying and capturing residents' spontaneous EoLC conversation triggers in daily life and giving positive feedback.	Theme 1–2
		Assessing residents' palliative care needs timely and introducing palliation of suffering when residents express a desire for a painless death.	Theme 1–3
		Acting as a third party to build bridges between residents and their families to facilitate effective communication around EoLC.	Theme 1–2
		The information sharing should be driven by truth to develop trusting relationships with residents and family members.	Theme 3–1
Individual	Healthcare providers	Assisting residents in making decisions about current treatment and care, rather than focusing attention on future.	Theme 2–1; 2–3
		Incorporating non‐medical considerations (i.e. social, spiritual and psychological needs) into ACP conversations.	Theme 4–2
		Revisiting ACP to reflect the iterative nature of decision‐making that occurs along residents' changeable physical and mental conditions and preferences, rather than a static event.	Theme 2–3

Abbreviations: ACP, advance care planning; EoLC, end‐of‐life care; LTCFs, long‐term care facilities.

## 6 CONCLUSION

This study provides a detailed understanding of potential barriers and facilitators to advance care planning in long‐term care facilities in mainland China, which fill the gaps identified in our realist review. The findings also describe how complex and relational end‐of‐life decision‐making processes in long‐term care facilities may complicate advance care planning. Our evidence‐based recommendations can inform the development and implementation of context‐specific advance care planning programmes in mainland China and other Asia‐Pacific regions with similar cultural contexts. Further research should focus on exploring and comparing perceptions and preferences on advance care planning among stakeholders in government‐operated long‐term care facilities and rural areas.

## AUTHOR CONTRIBUTIONS

Yuxin Zhou: Conceptualisation, methodology, investigation, formal analysis, data curation, writing—original draft, writing—review and editing and project administration. Ariel Wang: Validation, formal analysis and writing—review and editing. Debbie Braybrook: Conceptualisation, formal analysis, writing—review and editing and supervision. Clare Ellis‐Smith: Conceptualisation, formal analysis, writing—review and editing and supervision. Haixia Feng: Validation, investigation and writing—review and editing. Ni Gong: Validation, formal analysis and writing—review and editing. Zhi Zhou: Validation, investigation and writing—review and editing. Richard Harding: Conceptualisation, formal analysis, writing—review and editing and supervision.

## FUNDING INFORMATION

This work was supported by Guangzhou Concord Medical Humanities Research and Education Fund [grant number 23000‐3050070] and the Horizontal Research Project of Zhongda Hospital Affiliated to Southeast University [grant number 2021040047].

## CONFLICT OF INTEREST STATEMENT

The authors declare that they have no known competing financial interests or personal relationships that could have appeared to influence the work reported in this paper.

## Supporting information


Data S1.

File S1.
File S2.
File S3.
File S4.

## Data Availability

The data that support the findings of this study are available on request from the corresponding author. The data are not publicly available due to privacy or ethical restrictions.
