# Supplementary material for: What are the barriers and facilitators to advance care planning with older people in long‐term care facilities? A qualitative study
Source: J Clin Nurs. 2024 Feb 20;35(5):2356–70. doi: 10.1111/jocn.17071 (PMC13068171; doi:10.1111/jocn.17071)
Supplement: Supplementary file 2 — File S1. File S2. File S3. File S4. [file JOCN-35-2356-s002.docx]

**Supplementary file 1. Consolidated criteria for reporting qualitative studies (COREQ): 32-item checklist**

| **Topic** | **Item No.** | **Guide questions/description** | **Reported on Page No.** |
| --- | --- | --- | --- |
| **Domain 1: Research team and reﬂexivity** | | | |
| *Personal Characteristics* | | | |
| Interviewer/facilitator | 1 | Which author/s conducted the interview or focus group? | 6 |
| Credentials | 2 | What were the researcher’s credentials? E.g. PhD, MD | 6 |
| Occupation | 3 | What was their occupation at the time of the study? | 6 |
| Gender | 4 | Was the researcher male or female? | 6 |
| Experience and training | 5 | What experience or training did the researcher have? | 6 |
| *Relationship with participants* | | | |
| Relationship established | 6 | Was a relationship established prior to study commencement? | 6 |
| Participant knowledge of the interviewer | 7 | What did the participants know about the researcher? e.g. personal goals, reasons for doing the research | 5-6 |
| Interviewer characteristics | 8 | What characteristics were reported about the interviewer/facilitator? e.g. Bias, assumptions, reasons and interests in the research topic | 6; 29 |
| **Domain 2: study design** | | | |
| *Theoretical framework* | | | |
| Methodological orientation and Theory | 9 | What methodological orientation was stated to underpin the study? e.g. grounded theory, discourse analysis, ethnography, phenomenology, content analysis | 5-7 |
| *Participant selection* | | | |
| Sampling | 10 | How were participants selected? e.g. purposive, convenience, consecutive, snowball | 5-6 |
| Method of approach | 11 | How were participants approached? e.g. face-to-face, telephone, mail, email | 6 |
| Sample size | 12 | How many participants were in the study? | 9-11 |
| Non-participation | 13 | How many people refused to participate or dropped out? Reasons? | 9 |
| *Setting* | | | |
| Setting of data collection | 14 | Where was the data collected? e.g. home, clinic, workplace | 5 |
| Presence of non-participants | 15 | Was anyone else present besides the participants and researchers? | N/A |
| Description of sample | 16 | What are the important characteristics of the sample? e.g. demographic data, date | 10-11 |
| *Data collection* | | | |
| Interview guide | 17 | Were questions, prompts, guides provided by the authors? Was it pilot tested? | 6-7; supplementary file 2-3 |
| Repeat interviews | 18 | Were repeat interviews carried out? If yes, how many? | No |
| Audio/visual recording | 19 | Did the research use audio or visual recording to collect the data? | 7 |
| Field notes | 20 | Were ﬁeld notes made during and/or after the interview or focus group? | 7 |
| Duration | 21 | What was the duration of the interviews or focus group? | 9 |
| Data saturation | 22 | Was data saturation discussed? | 5-6 |
| Transcripts returned | 23 | Were transcripts returned to participants for comment and/or correction? | No |
| **Domain 3: analysis and findings** | | | |
| *Data analysis* | | | |
| Number of data coders | 24 | How many data coders coded the data? | 7-8 |
| Description of the coding tree | 25 | Did authors provide a description of the coding tree? | 12-24 |
| Derivation of themes | 26 | Were themes identiﬁed in advance or derived from the data? | 7 |
| Software | 27 | What software, if applicable, was used to manage the data? | 7 |
| Participant checking | 28 | Did participants provide feedback on the ﬁndings? | No |
| *Reporting* | | | |
| Quotations presented | 29 | Were participant quotations presented to illustrate the themes/ﬁndings? Was each quotation identiﬁed? e.g. participant number | 12-23 |
| Data and ﬁndings consistent | 30 | Was there consistency between the data presented and the ﬁndings? | 12-23 |
| Clarity of major themes | 31 | Were major themes clearly presented in the ﬁndings? | 12-23 |
| Clarity of minor themes | 32 | Is there a description of diverse cases or discussion of minor themes? | 12-23 |

Developed from: Tong A, Sainsbury P, Craig J. Consolidated criteria for reporting qualitative research (COREQ): a 32-item checklist for interviews and focus groups. *International Journal for Quality in Health Care*. 2007. Volume 19, Number 6: pp. 349 – 35

**Supplementary file 2. Interview topic guides**

**Qualitative Interview Topic Guide – Residents/Family members**

*Family members were asked the same questions but framed from their experiences with residents.

**Opening question:**

- “When and why you were admitted to the facility?”

- “How do you feel about the services/care you received here?”

| **Topic area 1: Explore residents’ experiences of end-of-life care communication** | |
| --- | --- |
| **Question area** | **Prompts** |
| Can you tell me more about your illness? | - What are the effects of illness on your daily life, including physical, psychological, social, and spiritual areas? |
| Of the things you have just spoken about, what matters to you most? | - Physical, psychological, social, and spiritual areas. |
| Have you had discussions with others about these things, and how you would like to be looked after at the end of life? | - What will motivate you to talk about your needs and choices with them? Like under what environment? With what relationships? Under what health status? |

**[Show Vignette one]**

| **Topic area 2: Explore residents’ experiences in making decisions on care at the end-of-life** | |
| --- | --- |
| **Question area** | **Prompts** |
| If you become very ill or in the last year of life like Mr Zhang, what sort of things do you think important to think about or plan for? | - Including physical, psychological, social, and spiritual areas.  - Where would you prefer to be treated? (home, hospital or nursing home?)  - Would you like to receive life-sustaining treatment? |
| Who do you think should make decisions or plans for Mr Zhang? | - Mr Zhang, his healthcare professionals or family? |
| How are decisions about your care usually made? | - Who initiates the discussion?  - Who makes the decisions? |
| If Mr Zhang became very ill towards the end of life or developed dementia, who do you think should make decisions on his behalf? | - healthcare professionals, family members or both?  - Do you think they could fully understand Mr Zhang’s needs and choices? |

**[Show Vignette two]**

| **Topic area 3: Introduce the concept of ACP and explore resident’ views and preferences for ACP** |
| --- |
| **Introduction:**  Following the vignette, we would like to introduce you to a new program called “*Advance Care Planning (ACP)*”. It is a communication process to enable patients to define their preferences and wishes for care. It also enabled them to discuss these preferences with their families and the healthcare professionals caring for them and to document, and review these, if appropriate. We hope to collect your opinions and attitudes towards ACP to ensure it is developed and implemented in an acceptable way in China. |

| **Question area** | **Prompts** |
| --- | --- |
| Can you tell me have you heard about/done ACP before? | - Where did you hear about it before?  - What have you heard about it? |
| Can you tell me how do you feel about having a communication to plan for and discuss the future, like in the vignette? | - Positive or negative? Why?  - Would you be willing to be involved in the communication where appropriate? Why?  - What factors affect your attitude? |
| What would be the benefits of having a communication? | - How do you think it might help? |
| What do you think the problems or challenges might be with having a communication? | - Can you think of anything that would help to overcome these challenges? |
| Can you tell me what you expect an ACP to be like? | - Who do you think should attend?  - What is the role of people attend?  - Who shall initiate the communication?  - What topics do you think would be important and should be discussed in such a communication?  - When is the best time to initiate a communication? And when is the best time to review it again?  - Would you prefer your decisions to be documented?  - Would it be helpful to receive some form of documentation that help you start thinking through your care plans or wishes?  - Who shall make the final decisions? |
| Would you like family members to attend a communication with you? | - What are the benefits and challenges of having family there? |
| What circumstances would make it easier for you to participate in such discussions? |  |
| What would make it more difficult for you to participate in such discussions? |  |

| **Additional questions** | |
| --- | --- |
| **Question area** | **Prompts** |
| Thank you, your answers are really helpful. Before we finish, is there anything else you would like to talk about? | How do you feel about having this conversation? |

**Qualitative Interview Topic Guide - Healthcare professionals**

**Opening question:**

- “Can you please tell me about your role in the medical team?”

| **Topic area 1: Explore healthcare professionals’ experiences of end-of-life care communication** | |
| --- | --- |
| **Question area** | **Prompts** |
| What illness do residents have in the facility? | - What are the effects of illness on their daily life, including physical, psychological, social, and spiritual areas? |
| Of the things you have just spoken about, what matters to residents most? | - Physical, psychological, social, and spiritual areas.  - How do you ascertain their needs? |
| Have you had discussions of end-of-life care with with residents and their relatives? How do you discuss such topics with them? | - Who would be the first person you discussed with?  - What specific topics are usually discussed?  - When would you initiate such discussions? |
| How do you feel about participating in planning and discussing care in the future or end-of-life care for residents? | - What are the benefits and challenges of planning or discussing the future with residents and relatives? |

**[Show Vignette one]**

| **Topic area 2: Explore healthcare professionals’ experiences in making decisions on care at the end-of-life with residents and/or families** | |
| --- | --- |
| **Question area** | **Prompts** |
| If residents become very ill or in the last year of life like Mr Zhang, what sort of things do you think are important for residents to think about or plan for? | - Including physical, psychological, social, and spiritual areas. |
| What are the challenges of delivering care that is aligned with residents’ needs? | - What circumstances would make it easier for you to deliver care that is aligned with residents' needs? |
| Who do you think should make decisions or plans for Mr Zhang? | - Mr Zhang, healthcare professionals or family? |
| How are decisions about care in residents’ critical medical situations or at the end of life usually made? | - Who did you discuss with?  - Who usually made the final decisions? |
| If Mr Zhang became very ill towards the end of life or developed dementia, who do you think should make decisions on his behalf? | - Healthcare professionals, family members or both?  - Do you think they could fully understand Mr Zhang’s needs and choices? |

**[Show Vignette two]**

| **Topic area 3: Introduce the concept of ACP and explore healthcare professionals’ views and preferences for ACP** | |
| --- | --- |
| **Introduction:**  Following the vignette, we would like to introduce you to a new program called “*Advance Care Planning (ACP)*”. It is a communication process to enable patients to define their preferences and wishes for care. It also enabled them to discuss these preferences with their families and the healthcare professionals caring for them and to document, and review these, if appropriate. We hope to collect your opinions and attitudes towards ACP to ensure it is developed and implemented in an acceptable way in China. | |
| **Question area** | **Prompts** |
| Can you tell me have you heard about/done ACP before? | - Where did you hear about it before?  - What have you heard about it? |
| Can you tell me how do you feel about having a communication to plan for and discuss the future, like in the vignette? | - Positive or negative? Why?  - Would you be willing to be involved in a communication where appropriate? Why?  - What factors affect your attitude? |
| What would be the benefits of having this communication? | - How do you think it might help? |
| What do you think the problems or challenges might be with having this communication? | - Can you think of anything that would help to overcome these challenges? |
| Can you tell me what you expect the communication to be like? | - Who do you think should attend?  - What is the role of people attend?  - Who shall initiate the communication?  - What topics do you think would be important and should be discussed in such communication?  - When is the best time to initiate the meeting? And when is the best time to review it again?  - Would you prefer your decisions to be documented?  - Would it be helpful to receive some form of documentation that help you start thinking through your care plans or wishes?  - Who shall make the final decisions? |
| Would you like family members attend the communication? | - What are the benefits and challenges of having family there? |
| What circumstances would make it easier for you to participate in such discussions? |  |
| What would make it more difficult for you to participate in such discussions? |  |
| Can you tell me how you feel about the implementation of ACP in China? | - Why?  - What factors do you think motivate or hinder the implementation of ACP in China?  - What challenges do you think will be faced in the implementation of ACP in China? How to address it? |

| **Additional questions** | |
| --- | --- |
| **Question area** | **Prompts** |
| Thank you, your answers are really helpful. Before we finish, is there anything else you would like to talk about? | How do you feel about having this conversation? |

**Supplementary file 3. Vignettes for interview**

**Vignette one**

| Mr Zhang is a 89 years old who has been moved to a nursing home for six months. As time goes by, Mr Zhang experienced an acute decline in his memory and had a chest infection, his doctor prescribed an antibiotic to control the infection. However, after 3 months, Mr Zhang’s breathing got worse. The doctor explained that he might not get better with the antibiotics, and suggested Mr Zhang receive trachea intubation to keep him safe.  However, Mr Zhang did not want to receive intubation at the end-of-life. He said: “I don’t like living with the tube, It’s not comfortable”. Additionally, there is conflict in the family. Mr Zhang’s son said his father needs the treatment, it was a filial duty to keep her father alive longer, so he persuaded Mr Zhang to receive the treatment; but the daughter thought the treatment will simply prolong her father’s life.  Mr Zhang was also afraid of developing dementia in the future, he had seen some of his friends who have developed dementia and had been unable to make decisions and express his preferences for end-of-life care anymore. |
| --- |

**Vignette two**

| Two weeks later:  Mr Zhang expressed his wishes to have a discussion with his healthcare provider and close family. His doctor arranged a meeting with Mr Zhang and his family to talk about his needs and care for the end-of-life. The doctor explained to Mr Zhang and his family what could happen if the chest infection process and encouraged Mr Zhang to express his preferences for treatment and care at the end of life, such as whether be willing to receive trachea intubation and his preferred healthcare decision maker in case he loses the capacity to make decisions in the future. Also, a personal booklet was provided to summarise Mr Zhang’s wishes and preferences and document his healthcare concerns. |
| --- |

**Supplementary file 4. Brief introduction to the legislation of the Living Will in Shenzhen, China**

In June 2022, the tenth meeting of the Standing Committee of the Seventh Shenzhen Municipal People's Congress voted and passed the revised draft of the "Shenzhen Special Economic Zone Medical Regulations", which will take effect in January 2023. The Action 78 of the "Shenzhen Special Economic Zone Medical Regulations" requires that medical institutions should respect the living will of patients who are at the terminal phase of an incurable disease or their end of life when taking medical treatment and care under the following circumstances. This is the first time in China that the patient's end-of-life decision right - living will was written into local regulations:

1. There is a clear statement of intention to take or not to take intubation, cardiopulmonary resuscitation and other traumatic resuscitation measures, to use or not to use life-sustaining treatments, and to perform or not to perform continuous treatment of the primary disease.
2. Notarised or witnessed by two or more witnesses, and the witnesses must not be healthcare professionals involved in the treatment of patients.
3. The living will is in the form of a written document or audio/video record. In addition to notarisation, the patient and the witnesses should sign and date the written document; The name or photo of the patient and the witness and the time should be recorded in the audio/video record.

Reference:

1. Shenzhen Municipal People’s Congress. Announcement of the Standing Committee of the Seventh Shenzhen Municipal People’s Congress (No.56). <http://www.szrd.gov.cn/rdlv/chwgg/content/post_826158.htm> [Accessed 2022-07-05].
